# *Caballeronia* Gut Symbionts in Species of the Seed Bug Family Heterogastridae (Heteroptera: Lygaeoidea)

**DOI:** 10.1264/jsme2.ME25061

**Published:** 2025-12-17

**Authors:** Antoine-Olivier Lirette, Kota Ishigami, Minhyung Jung, Yu Matsuura, Yoshitomo Kikuchi

**Affiliations:** 1 Graduate School of Agriculture, Hokkaido University, 060–8589 Sapporo, Japan; 2 Biomanufacturing Process Research Center, National Institute of Advanced Industrial Science and Technology (AIST), Hokkaido Center, 062–8517 Sapporo, Japan; 3 Entomological Laboratory, College of Agriculture, University of the Ryukyus, Nishihara, Okinawa 903–01, Japan; 4 Tropical Biosphere Research Center, University of the Ryukyus, Nishihara, Okinawa 903–01, Japan

**Keywords:** symbiosis, Heterogastridae, stinkbug, *Caballeronia*

## Abstract

Most phytophagous species of stinkbugs have mutualistic relationships with bacterial symbionts, which are often located within specialized midgut regions called M4. Heterogastridae, previously classified within the family Lygaeidae, are now classified as a family proper; however, the symbiotic organ’s morphology and symbiont identity remain unclear. We herein investigated symbiotic systems from two heterogastrid species. The results obtained show that they possess two rows of midgut crypts akin to those of Coreoidea and consistently associate with *Caballeronia* symbionts of the SBE-α and Coreoidea clades. The present study clearly demonstrates that *Caballeronia* bacteria are symbionts of Heterogastridae and is the first to report a Coreoidea clade symbiont from the superfamily Lygaeoidea.

Many phytophagous stinkbugs of the infraorder Pentatomomorpha commonly harbor symbiotic bacteria extracellularly in the lumen or sac-like tissues of their midgut called “crypts” (Kikuchi *et al.*, 2008; Prado and Zucchi, 2012). Members of the superfamilies Lygaeoidea and Coreoidea generally associate with a clade of *Betaproteobacteria* from‍ ‍the genus *Caballeronia*, each generation of which is acquired environmentally by nymphs (Kikuchi *et al.*, 2007, 2011). Due to their environmental transmission and *Caballeronia*’s free-living capacity, host and symbiont evolutionary histories do not mirror each other in these systems, unlike many insect-bacteria symbioses (Kikuchi *et al.*, 2008, 2009). The symbiotic organ’s morphology differs across taxa, with long tubular outgrowths being reported in the superfamily Lygaeoidea and two rows of crypts in the superfamily Coreoidea (Kikuchi *et al.*, 2011). However, the family Pachygronthidae, a basal family of the superfamily Lygaeoidea, which represents one of the earliest diverging lineages within the clade and is, thus, closer to their ancestor (Zhang *et al.*, 2024), has Coreoidea-like crypts (Kikuchi *et al.*, 2011). Therefore, the symbiotic system of intermediate taxa between the two superfamilies may offer insights into the evolution of stinkbug-*Caballeronia* symbiosis.

The family Heterogastridae, formerly a subfamily of the family Lygaeidae, consists of ~100 species (Henry, 1997; Cassis and Gross, 2002) in the superfamily Lygaeoidea from‍ ‍which they diverged early alongside the family Pachygronthidae (Zhang *et al.*, 2024). Despite their symbiotic system’s potential importance in understanding stinkbug-*Caballeronia* symbiosis, it remains largely unknown. Therefore, the present study investigated the phylo­genetic positions of two Heterogastridae species, *Heterogaster urticae* and *Sadoletus valdezi* (Fig. 1A and B) as well as their symbiotic bacteria by sequencing mitochondrial cytochrome c oxidase subunit I (COI) of the insects and 16S ribosomal RNA (16S rRNA) genes of the bacterial symbionts.

Specimens of *H. urticae* and *S. valdezi* were collected from Hokkaido and Okinawa, Japan (Table S1), and were brought to the laboratory either alive or preserved in acetone. Through dissection under a stereomicroscope (M205 FA; Leica), we confirmed that both species possessed a distinct white midgut section, which presumably serves as the symbiotic organ. This organ forms the 4^th^ midgut region (M4), which is connected to the 3^rd^ region (M3) by an anterior crypt-free “bulb” (M4B) (Fig. 1C and D), and possesses two rows of crypts along its central lumen (Fig. 1C and D). M3 and M4B are connected by a narrow region resembling the “constricted region” (CR) of *Riptortus pedestris*, a member of the superfamily Coreoidea, which is involved in symbiont sorting (Ohbayashi *et al.*, 2015).

To identify the phylogenetic placement of the insect species, their mitochondrial COI genes were sequenced with the invertebrate universal primer set, LCO1490 and HCO2198 (Folmer *et al.*, 1994), under previously reported conditions (Morimura *et al.*, 2024). Phylogenetic analyses were performed using MEGA11 v11.0.13 (Tamura *et al.*, 2021); alignment with related heteropteran sequences obtained from NCBI was conducted using CLUSTAL W (Thompson *et al.*, 1994) and a maximum likelihood phylogenetic tree was constructed with its robustness assessed by bootstrapping (Tamura-Nei model, 1,000 bootstrap replicates). Based on the COI gene, we confirmed that our Heterogastridae stinkbugs formed the basal lineage of the superfamily Lygaeoidea alongside the family Pachygronthidae (Fig. 1E).

To identify the symbiotic bacteria of the two Hetero­gastridae species, we extracted the M4 region of adult *H. urticae* and *S. valdezi*, which is presumed to serve as the symbiotic organ. The extracted organ was washed with filter-sterilized phosphate-buffered saline (PBS) and then subjected to DNA extraction as described above. Bacterial 16S rRNA genes were then amplified using the universal primers 16SA1 and 16SB1 (Fukatsu and Nikoh, 1998), and PCR products were cleaned and sequenced with an ABI 3130xl DNA sequencer (Applied Biosystems). Nineteen high-quality 16S rRNA sequences were obtained from *H. urticae* and 6 from *S. valdezi*. Based on EzBioCloud as the 16S rRNA reference database (Chalita *et al.*, 2024), all sequences showed high homology (97.92–99.14%) with *Caballeronia* strains, most of which were too low to identify at the species level. Their identity was further clarified through a phylogenetic anal­ysis in which our sequences all clustered within the genus *Caballeronia* with high support values. Furthermore, all sequences analyzed by Sanger sequencing were successfully read without any cloning procedure (*i.e.*, direct sequencing), suggesting a simple microbiota and potential *Caballeronia* monoclonization of the M4 region. The phylogenetic anal­ysis of 16S rRNA, performed using the same method as that for the host COI anal­ysis, showed that the symbiotic bacteria of *H. urticae* and *S. valdezi* belonged to three distinct clades of *Caballeronia* (Fig. 2). The *Caballeronia* genus is subdivided into 4 clades: the SBE-α, SBE-β, SBE-γ, and Coreoidea clades (Ohbayashi *et al.*, 2022). The 6 sequences identified from *S. valdezi* were placed in the SBE-α clade. Nine sequences obtained from *H. urticae* collected in Minami ward and 8 from Kiyota ward were placed within the Coreoidea clade, while the other two clustered with the SBE-γ clade.

To confirm the localization of the symbiont, diagnostic PCR was performed on each gut region and reproductive organs of three female *H. urticae* using the *Burkholderia sensu lato*-specific primers Burk16SF and Burk16SR (Kikuchi *et al.*, 2005). Gel electrophoresis results indicated that *Caballeronia* consistently inhabited M4, while its presence was more moderate in M3 (Fig. S1). The same diagnostic PCR was performed on 20 adult *H. urticae* and 50 *S. valdezi*, and all insects tested positive, except for one *S. valdezi* specimen, suggesting the universal prevalence of *Caballeronia* in the adult population.

Although the role of *Caballeronia* in Heterogastridae has yet to be investigated, their well-developed symbiotic organ populated with bacteria that are mostly absent from other gut regions strongly suggests a stable and specific, potentially positive, relationship, similar to that reported in other *Caballeronia*-stinkbug symbioses (Kikuchi *et al.*, 2007; Ohbayashi *et al.*, 2022; Ishigami *et al.*, 2023). The symbionts clustered with the SBE-α, SBE-γ, and Coreoidea clades. Although SBE-α is ubiquitous in both coreoid and lygaeoid stinkbugs and SBE-γ is found in Lygaeoidea (Kikuchi *et al.*, 2011; Ohbayashi *et al.*, 2022; Ishigami *et al.*, 2023), the Coreoidea clade has, until now, only been identified as symbionts of Coreoidea (Kikuchi *et al.*, 2011; Ohbayashi *et al.*, 2022). This represents the first example of a member of the Coreoidea clade being identified outside a species of the superfamily Coreoidea, indicating a relationship with other superfamilies.

Heterogastridae forms the basal lineage of the superfamily Lygaeoidea with its sister family Pachygronthidae (Fig. 1E; Zhang *et al.*, 2024). Considering the morphology of their symbiotic organs and the lineage of their symbiotic bacteria, Heterogastridae stinkbugs possess very similar characteristics to members of the superfamily Coreoidea, with two rows of crypts along a central lumen, filled with *Caballeronia* bacteria from clades strongly, or uniquely, associated with Coreoidea hosts (Kikuchi *et al.*, 2011; Kuechler *et al.*, 2016; Ohbayashi *et al.*, 2022) (also see Fig. 2). These features are also present in Pachygronthidae (Kikuchi *et al.*, 2011; Kang *et al.*, 2019), further supporting the present results, suggesting that the midgut symbiotic organ’s arrangement of two rows of crypts is ancestral in Coreoidea and Lygaeoidea. Members of the superfamily Pentatomoidea also commonly possess small crypts (Kikuchi *et al.*, 2011); therefore, this conformation may be the ancestral trait of the gut symbiotic organs of the infraorder Pentatomomorpha, although members of Pentatomoidea associated with *Gammaproteobacteria* symbionts and not *Caballeronia* (Fig. S2).

Although further research is needed to obtain a more detailed understanding of the role and diversity of *Caballeronia* symbionts in the family Heterogastridae, *in vivo* localization patterns, phylogenetic anal­yses, and the gut morphological characteristics of Heterogastridae provide compelling evidence of their symbiotic relationship and insights into the evolutionary process of symbiosis between Pentatomomorpha stinkbugs and *Caballeronia*.

## Data availability

All sequences of bacterial 16S rRNA and insect COI genes obtained in this study have been deposited in NCBI and DDBJ under accession numbers PV444344–PV444358 and PX232669–PX232678, and LC867680–LC867685, respectively.

## Citation

Lirette, A.-O., Ishigami, K., Jung, M., Matsuura, Y., and Kikuchi, Y. (2025) *Caballeronia* Gut Symbionts in Species of the Seed Bug Family Heterogastridae (Heteroptera: Lygaeoidea). *Microbes Environ ***40**: ME25061.

https://doi.org/10.1264/jsme2.ME25061

## Supplementary Material

Supplementary Material

## Figures and Tables

**Fig. 1.**
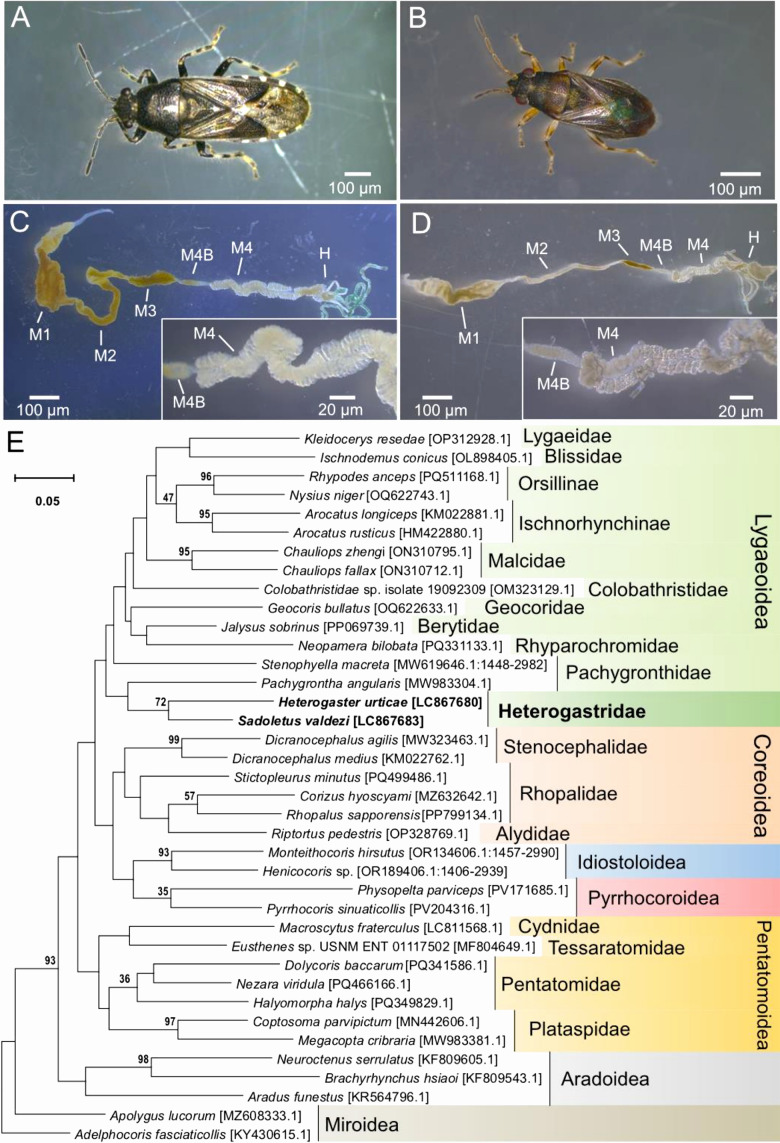
Species of the family Heterogastridae collected from Japan and their phylogenetic position in the stinkbug infraorder Pentatomomorpha. Adult female (A) *Heterogaster urticae* and (B) *Sadoletus valdezi* and their midguts (C and D). Midgut sections: M1, midgut first section; M2, midgut second section; M3, midgut third section; M4, midgut fourth section; M4B, M4 bulb; CR, constricted region; H, hindgut. The insets show the crypt arrangement in both species. (E) A maximum likelihood tree of the infraorder Pentatomomorpha, based on 650‍ ‍bp of mitochondrial COI gene sequences. Supporting values (1,000 bootstrap replicates) more than 30% are depicted on the tree nodes. Heterogastridae species are shown in dark green. Accession numbers are shown in brackets.

**Fig. 2.**
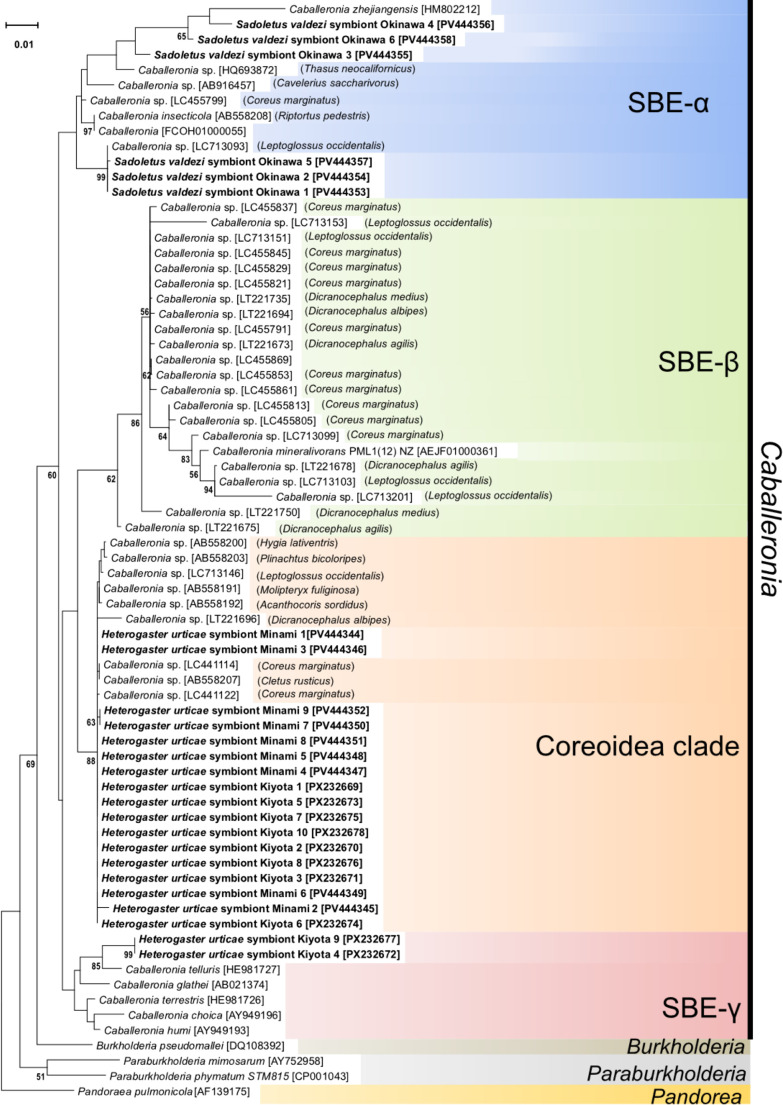
Phylogenetic position of *Caballeronia* symbionts detected in *Heterogaster urticae* and *Sadoletus valdezi*. A maximum likelihood tree based on 1,400‍ ‍bp of 16S rRNA gene sequences. Three representative symbionts of both Heterogastridae species are shown. Subclades of the genus *Caballeronia* (the SBE-α, SBE-β, SBE-γ, and Coreoidea clades) are shown on the right side. Supporting values (1,000 bootstrap replicates) >50% are depicted on the tree nodes. Accession numbers are shown in brackets. Hosts of symbiotic strains are shown in parentheses.
