# Supplementary material for: Caballeronia Gut Symbionts in Species of the Seed Bug Family Heterogastridae (Heteroptera: Lygaeoidea)
Source: Microbes Environ. 2025 Dec 17;40(4):ME25061. doi: 10.1264/jsme2.ME25061 (PMC12727193; doi:10.1264/jsme2.ME25061)
Supplement: Supplementary file 1 — Supplementary Material [file 40_25061_s1.pdf]

Table S1. Sampling Sites Information. Location and date of collection of the insect samples, experiment they were involved with, and the accession numbers of host species' COI genes and associated symbionts 16S rRNA genes used in the phylogenetic trees.

Species	Location	Date	Host Plant	Experiment	Host Accession	Symbiont Accession
Sadoletus valdezi	Motobu, Kunigami, Okinawa, Japan	3.8.2024	*Ficus superba*	Host and symbiont sequencing	LC867683-LC867685	PV444353-PV444358
Heterogaster urticae	Minami-ku, Sapporo, Hokkaido, Japan	14.7.2021	*Urtica thunbergiana*	Host and symbiont sequencing	LC867680-LC867682	PV444344-PV444352
Heterogaster urticae	Kiyota-ku, Sapporo, Hokkaido, Japan	20.7.2024	*Urtica thunbergiana*	Symbiont sequencing and rearing experiment		PX232669-PX232678
Heterogaster urticae	Kiyota-ku, Sapporo, Hokkaido, Japan	7.6.2025	*Urtica thunbergiana*	Gut region diagnostic PCR		

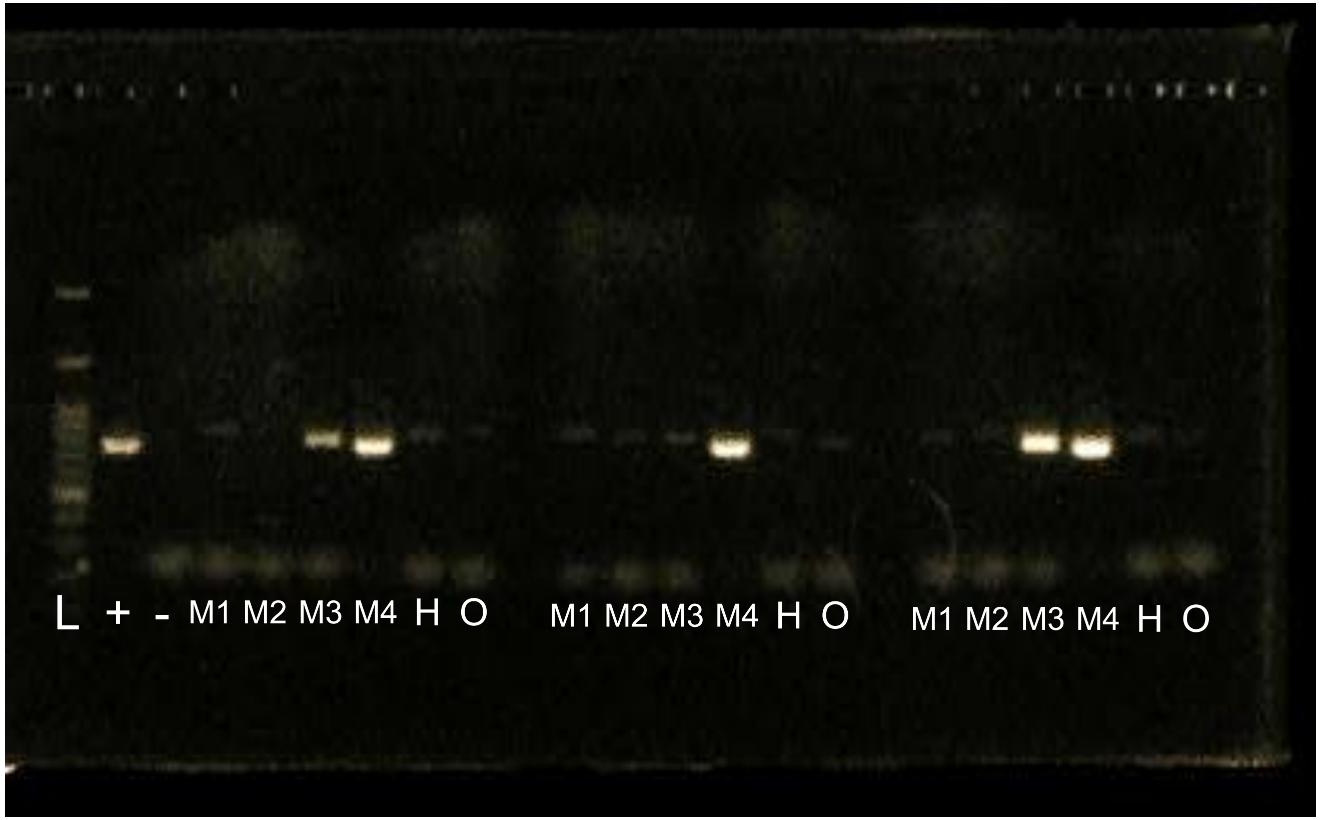

Figure S1. Gut Regions Diagnostic PCR
Gel electrophoresis of diagnostic PCR results for 3 female *H. urticae.* L: ladder; +: positive control; -: negative control; M1, midgut first section; M2, midgut second section; M3, midgut third section; M4, midgut fourth section; H, hindgut; O: Ovaries. In this experiment, universal primers for *Burkholderia sensu lato* were used, and since it is thought that a variety of bacteria inhabit the M3 region, it is possible that PCR amplification was observed that was different from the target *Caballeronia* symbiotic bacteria.

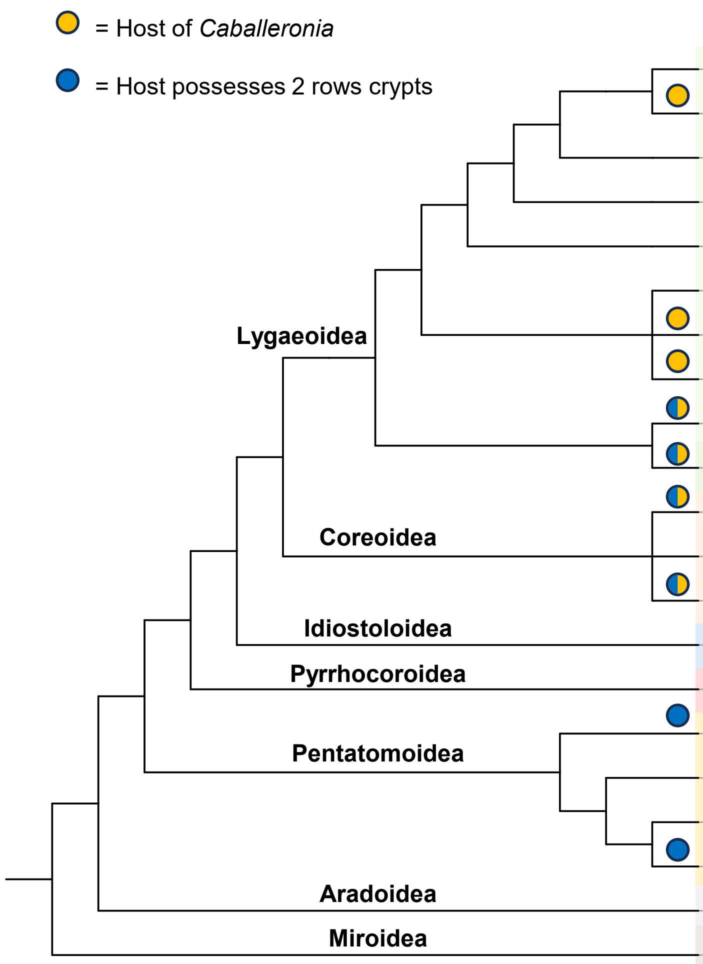

Family	Symbiont [a]	Symbiotic Organ Type [b]	Feeding Type [c]	Source
Lygaeidae	Enterobacteriaceae	Bacteriome	Phyto	Kuechler et al., 2012
Blissidae	*Caballeronia sp.* (SBE-α) or *Ischnodemia sp.*	Tubes or bacteriome	Phyto	Kikuchi et al. 2011; Kuechler et al., 2012
Orsillinae	*Schneideria sp.*	bacteriome	Phyto	Matsuura et al., 2012
Malcidae	No symbiont	No symbiotic organ	Phyto	Kikuchi et al., 2011
Colobastridae	unknown	unknown	Phyto	-
Geocoridae	No symbiont	No symbiotic organ	Pred	Kikuchi et al., 2011; Kuechler et al., 2012
Berytidae	*Caballeronia sp.* (SBE-α)	Tubes	Phyto	Kikuchi et al., 2011
Rhyparochromidae	*Caballeronia sp.* (SBE-α)	Tubes	Phyto	Kikuchi et al., 2011
Pachygronthidae	*Caballeronia sp.* (SBE-α)	2 rows crypts	Phyto	Kikuchi et al., 2011
Heterogastridae	*Caballeronia sp.* (SBE-α, SBE-γ, Coreoidea-clade)	2 rows crypts	Phyto	This Study
Stenocephalidae	*Caballeronia sp.* (SBE-α, SBE-β, Coreoidea-clade)	2 rows crypts	Phyto	Kuechler et al., 2016
Rhopalidae	No symbiont	No symbiotic organ	Phyto	Kikuchi et al., 2011
Alydidae	*Caballeronia sp.* (SBE-α)	2 rows crypts	Phyto	Kikuchi et al. 2011
Idiostoloidae	unknown	unknown	Phyto	-
Pyrrhocoridae	Diverse	enlarged M3	Phyto	Sudakaran et al., 2015
Cydnidae	Enterobacteriaceae	2 rows crypts	Phyto	Hosokawa et al., 2012; Nakawaki et al., 2024
Tessaratomidae	unknown	unknown	Phyto	Liu et al., 2022; Schwertner et al., 2015
Pentatomidae	*Pantoea sp.*	4 rows crypts	Phyto	Bansal et al., 2014
Plataspidae	*Ishikawaella sp.*	2 rows crypts	Phyto	Hosokawa et al., 2006
Aradidae	No symbiont	No symbiotic organ	Myco	Kikuchi et al., 2011
Miridae	No symbiont	No symbiotic organ	Phyto	Kuechler et al., 2012; Weirauch et al., 2019

Figure S2. Summary Figure of Host-Symbiont Associations

Phylogenetic relationships of stinkbug families in infraorder Pentatomomorpha. The tree is based on our phylogenetic tree of host COI gene sequences. The name of the superfamilies is indicated in bold over their respective branch. The symbiont associated with each family, their symbiotic organ type, and their main feeding type are indicated in the table on the right. The clade of *Caballeronia* symbionts associated with each family is indicated in parentheses based on our phylogenetic tree of symbiont 16S rRNA gene sequences. An orange circle over a family's branch indicates hosts which commonly form symbiotic relationships with *Caballeronia* symbionts, a blue circle indicates hosts possessing 2 rows of crypts along their midgut 4th section as symbiotic organ. A circle with both colours indicates both features are present in the family. [a] Diverse: Gut microbiota consisting of Actinobacteria, Firmicutes, and γ-Proteobacteria; [b] Tubes: Tubular crypts along the midgut 4th section; 2 rows crypts: Two rows of small crypts along the midgut 4th section; 4 rows crypts: Four rows of small crypts along the midgut 4th section. [c] Phyto: phytophagous; Pred: predacious; Myco: mycophagous.

References of Figure S2.

1. Bansal, R., Michel, A. P., & Sabree, Z. L. (2014). The Crypt-Dwelling Primary Bacterial Symbiont of the Polyphagous Pentatomid Pest Halyomorpha halys (Hemiptera: Pentatomidae). Environmental Entomology, 43(3), 617–625. https://doi.org/10.1603/EN13341
2. Hosokawa, T., Kikuchi, Y., Nikoh, N., Shimada, M., & Fukatsu, T. (2006). Strict Host-Symbiont Cospeciation and Reductive Genome Evolution in Insect Gut Bacteria. PLoS Biology, 4(10), e337. https://doi.org/10.1371/journal.pbio.0040337
3. Hosokawa, T., Kikuchi, Y., Nikoh, N., & Fukatsu, T. (2012). Polyphyly of Gut Symbionts in Stinkbugs of the Family Cydnidae. Applied and Environmental Microbiology, 78(13), 4758–4761. https://doi.org/10.1128/AEM.00867-12
4. Kikuchi, Y., Hosokawa, T., & Fukatsu, T. (2011). An ancient but promiscuous host–symbiont association between Burkholderia gut symbionts and their heteropteran hosts. The ISME Journal, 5(3), 446–460. https://doi.org/10.1038/ismej.2010.150
5. Kuechler, S. M., Renz, P., Dettner, K., & Kehl, S. (2012). Diversity of Symbiotic Organs and Bacterial Endosymbionts of Lygaeoid Bugs of the Families Blissidae and Lygaeidae (Hemiptera: Heteroptera: Lygaeoidea). Applied and Environmental Microbiology, 78(8), 2648–2659. https://doi.org/10.1128/AEM.07191-11
6. Kuechler, S. M., Matsuura, Y., Dettner, K., & Kikuchi, Y. (2016). Phylogenetically Diverse Burkholderia Associated with Midgut Crypts of Spurge Bugs, Dicranocephalus spp. (Heteroptera: Stenocephalidae). Microbes and Environments, 31(2), 145–153. https://doi.org/10.1264/jsme2.ME16042
7. Liu, Z.-H., Yang, Z.-W., Zhang, J., Luo, J.-Y., Men, Y., Wang, Y., & Xie, Q. (2022). Stage correlation of symbiotic bacterial community and function in the development of litchi bugs (Hemiptera: Tessaratomidae). Antonie van Leeuwenhoek, 115(1), 125–139. https://doi.org/10.1007/s10482-021-01685-6
8. Matsuura, Y., Kikuchi, Y., Hosokawa, T., Koga, R., Meng, X.-Y., Kamagata, Y., Nikoh, N., & Fukatsu, T. (2012). Evolution of symbiotic organs and endosymbionts in lygaeid stinkbugs. The ISME Journal, 6(2), 397–409. https://doi.org/10.1038/ismej.2011.103
9. Nakawaki, T., Watanabe, S., & Hosokawa, T. (2024). The burrower bug Macroscytus japonensis (Hemiptera: Cydnidae) acquires obligate symbiotic bacteria from the environment. Zoological Letters, 10(1), 15. https://doi.org/10.1186/s40851-024-00238-9
10. Schwertner, C. F., & Grazia, J. (2015). Less diverse pentatomoid families (Acanthosomatidae, Canopidae, Dinidoridae, Megarididae, Phloeidae, and Tessaratomidae). In True bugs (Heteroptera) of the Neotropics (pp. 821-862). Dordrecht: Springer Netherlands.
11. Sudakaran, S., Retz, F., Kikuchi, Y., Kost, C., & Kaltenpoth, M. (2015). Evolutionary transition in symbiotic syndromes enabled diversification of phytophagous insects on an imbalanced diet. The ISME Journal, 9(12), 2587–2604. https://doi.org/10.1038/ismej.2015.75
12. Sudakaran, S., Retz, F., Kikuchi, Y., Kost, C., & Kaltenpoth, M. (2015). Evolutionary transition in symbiotic syndromes enabled diversification of phytophagous insects on an imbalanced diet. The ISME Journal, 9(12), 2587–2604. https://doi.org/10.1038/ismej.2015.75
